# Factors Contributing to the Delay in Diagnosis and Continued Transmission of Leprosy in Brazil – An Explorative, Quantitative, Questionnaire Based Study

**DOI:** 10.1371/journal.pntd.0004542

**Published:** 2016-03-15

**Authors:** Mary Henry, Noêmi GalAn, Katherine Teasdale, Renata Prado, Harpreet Amar, Marina S. Rays, Lesley Roberts, Pedro Siqueira, Gilles de Wildt, Marcos Virmond, Pranab K. Das

**Affiliations:** 1 Population Science and Humanities, University of Birmingham, Birmingham, United Kingdom; 2 Instituto Lauro de Souza Lima, Bauru, São Paulo, Brazil; 3 Faculdade de Medicina de Marilia (FAMEMA), Marilia, São Paulo, Brazil; 4 Centro Referencia em Tuberculose e Hanseniase, Sinop, Mato Grosso, Brazil; 5 Department of Clinical Immunology, School of Infection and Immunity, University of Birmingham, Birmingham, United Kingdom; University of Tennessee, UNITED STATES

## Abstract

**Background:**

Leprosy is a leading cause of preventable disability worldwide. Delay in diagnosis of patients augments the transmission of infection, and allows progression of disease and more severe disability. Delays in diagnosis greater than ten years have been reported in Brazil. To reduce this delay, it is important to identify factors that hinder patients from presenting to doctors, and those that delay doctors from diagnosing patients once they have presented. This study aimed to explore factors associated with the delayed diagnosis of leprosy in Brazil.

**Methodology/ Principal Findings:**

This is an exploratory study using a self-constructed questionnaire delivered to patients attending three leprosy referral clinics across three states in Brazil. Data were analysed to determine associations between variables and the time taken for participants to present to the health-service, and between variables and the time taken for doctors to diagnose participants once they had presented. Participants who suspected they had leprosy but feared community isolation were 10 times more likely to wait longer before consulting a doctor for their symptoms (OR 10.37, 95% CI 2.18–49.45, p = 0.003). Participants who thought their symptoms were not serious had a threefold greater chance of waiting longer before consulting than those who did (OR 3.114, 95% CI 1.235–7.856, p = 0.016). Forty-two point six per cent of participants reported initially receiving a diagnosis besides leprosy. These had a three times greater chance of receiving a later diagnosis of leprosy compared to those not misdiagnosed or not given a diagnosis (OR 2.867, 95% CI 1.288–6.384, p = 0.010).

**Conclusions/ Significance:**

This study implies a need for patient education regarding leprosy symptoms and the reduction of stigma to encourage patients to present. The high rate of misdiagnosis reported suggests a need to increase clinician suspicion of leprosy. Further education regarding disease symptoms in medical school curriculums may be advisable.

## Author Summary

Leprosy is caused by a bacterium that affects people’s nerves, giving rise to deformities. Leprosy largely affects the poor, and many people around the world are disabled due to leprosy. The deformities that the disease causes are largely preventable through early detection of the disease. In countries such as Brazil, where high rates of leprosy cases still exist, this disease remains a public health issue. However, long delays in the diagnosis of leprosy patients have been reported. In this study we observed that many patients were ignorant to the early symptoms of leprosy, and that the negative image surrounding leprosy within communities can hinder patients from seeking treatment due to the fear of being isolated. In addition, we noted that doctors often do not reach a conclusive diagnosis of leprosy promptly. This misdiagnosis appears to be contributing to the overall delay in the diagnosis of leprosy patients in areas of Brazil. Although more research exploring this subject should be conducted, this study highlights a possible need for further educational interventions for both patients and doctors.

## Introduction

Leprosy (Hansen’s disease) is one of the world’s Neglected Tropical Diseases and is recognised as ‘a disease of the poor’[1–3]. It is an infectious disease that is caused by the bacterium *Mycobacterium leprae*. This bacterium affects peripheral nerves and gives rise to deformities such as muscle wasting and injuries over anaesthetised areas of the body [2,4,5]. Leprosy is a leading cause of preventable disability, leaving 3 million people disabled worldwide [1]. Although much work has been done in recent years to eliminate the disease, leprosy remains endemic in nine counties, including Brazil. Brazil contributed 33 955 new cases in 2011, 16% of all new registered global cases, second only to India [6]. Furthermore, Brazil was not predicted to achieve the WHO’s ‘Final push’ goal for 2015. This was to reduce the number of new cases with grade 2 disability that were detected in 2010 by 35% (grade 2 disability is defined as visible damage or deformity) [7–9].

In order to break the cycle of transmission and reduce the number of new cases detected with physical deformity, it is essential to diagnose and treat patients early, before these injuries occur [5,8].

However, significant delays in diagnosis of leprosy have been reported in Brazil [10, 11]. These delays of up to 10 years, are unexpectedly even longer than those observed in some non-endemic countries [11]. The only study found in the literature search which explored reasons for the delay in diagnosis in Brazil, was an English language study from 1997 [10]. However, this study yielded no significant results and was limited to a small sample size of 40 participants; leaving much further need for research in this area.

We therefore conducted our study with the aim of exploring factors that contribute to the overall delay in diagnosis of leprosy. Such factors can be divided into two categories: those contributing to ‘patient delay’ (defined as the time between symptom onset and patients consulting a medical doctor) and those contributing to ‘health-system delay’ (defined as the time between first consulting a medical doctor and receiving a diagnosis of leprosy). Our study aimed to explore patient and health system delays separately; interested in identifying whether the delay in diagnosis of leprosy is associated with patients not presenting to the health service early enough or whether diagnosis is being delayed by the health service itself.

Literature from other countries has largely focused on patient delay. Demographic variables such as distance from the nearest health clinic and patients’ highest level of education were found to be associated with delay in China and India [12,13]. Additionally, leprosy patients have been found to prefer visiting traditional healers, rather than trained medical doctors [12,14,15]. As every society considers life and disease in different ways, it is essential to study similar questions regarding this delay within Brazil [16]. By exploring these demographic factors we can identify potential target groups for future public health interventions. Furthermore, the fear and stigma associated with leprosy in both endemic and non-endemic countries has been suggested to prevent patients from presenting to the health service for their symptoms [17–23]. This study aims to explore the effect of stigma through quantitative methods.

Leprosy care was integrated into primary care in Brazil in the year 2000 [24]. Although this may have encouraged patients to seek treatment, it is possible that clinical expertise regarding leprosy has declined. Health professionals may require more education regarding the early symptoms of leprosy, as has been suggested for tuberculosis [25]. Therefore, this study also aims to explore clinician suspicion of leprosy [13].

With previous literature in mind, this study was performed focusing on demographic variables, stigma, traditional healers, clinical examination and misdiagnosis, with the secondary aim of identifying factors that motivate patients to consult a medical doctor for their symptoms [13].

## Methods

### Setting

The primary setting for this research was *Lauro de Souza Lima Institute* (ILSL)–São Paulo—Brazil. This is a leprosy research and referral clinic, a governmental institute under the ministry of public health. ILSL serves a population of 350, 000 patients from all five geographical regions of Brazil. From here we visited two other referral centres to gain a larger sample size: *Sinop Reference Centre for Leprosy and Tuberculosis* (CRTHS)*—*Mato Grosso and *Dourados Reference Centre for Leprosy and Tuberculosis* (CRTHD)*—*Mato Grosso do Sul [26–28]. Participants attending these referral clinics during the study recruitment period were therefore not necessarily diagnosed there. Data collection and the study method were standardised throughout the three sites, with the same measurement tools and researchers in each. Referral clinics were chosen instead of primary care clinics, as the bases for this research in order to recruit sufficient numbers in the allocated time frame.

### Recruitment of participants

ILSL serves outpatients, inpatients, and residents of the former leprosy colony. All inpatients and residents were asked to participate. All leprosy patients attending outpatient appointments were also approached. Clinicians caring for these patients were first asked if researchers could inform their patients of the study.

Fifty-six participants were recruited from ILSL: 43 outpatients, 7 inpatients and 6 residents. All patients attending outpatient appointments at CRTHS and CRTHD were also asked to participate. Forty-two outpatients were recruited from Sinop and 24 from Dourados.

### Ethical considerations

Ethical approval from University of Birmingham BMedSc Population Sciences and Humanities internal ethics review and the Committee for Ethics in Research, Instituto Lauro de Souza Lima, Bauru, Brazil were obtained before commencing the study.

Adults previously diagnosed with leprosy either clinically or by laboratory testing were included in our study. Informed consent was gained from all participants. Written consent was gained from literate participants and verbal consent from illiterate participants. For literate participants unable to sign due to hand or finger deformities, consent was obtained via fingerprint. Prior approval for documenting oral and consent by fingerprint was obtained from the internal review board. After consulting with a member of the Brazilian Committee for ethics and medical director of ILSL, it was deemed culturally appropriate to accept the adult age for leprosy research to be 15 and over [8, 29]. Participants between 15 and 18 years of age consented for themselves, as they were competent and so consent was not sought from parents on their behalf.

### Exclusion criteria applied

1. Children below the age of 15 (as is culturally appropriate in Brazil) [8,29].
2. Adults lacking capacity to consent.
3. Patients diagnosed less than one month prior to their appointment. (To avoid possibly distressing newly diagnosed patients, who were still coming to terms with their diagnosis, by asking them to discuss events surrounding their diagnosis.)
4. Patients experiencing recent, serious life events (e.g. bereavement) or serious health problems at the time of interview (e.g. leprosy reaction). (This was done as a sensitive precaution to ensure participants were both physically and mentally able to provide accurate information.)

### Study design

This exploratory study used a self-constructed, quantitative questionnaire (S1 Appendix), which was delivered to participants over a seven-week recruitment period in February/March 2014. Literate participants completed the questionnaire themselves, whilst an interpreter delivered the questionnaire verbally to illiterate participants and those unable to write due to pain or hand deformities.

As this was an explorative study, maximum feasibility sampling was used. This study was part of a wider study that involved co-researchers.

### Measurement tool

No validated questionnaire relating to the subject was available; therefore, questions were formulated based on findings from previous qualitative and quantitative studies in Brazil and elsewhere [11–15,30–32]. The questionnaire was translated into Portuguese and piloted on consenting patients at ILSL. Issues with understanding were identified and the questionnaire adjusted accordingly.

In the questionnaire, participants were asked to report the time elapsed between first noting leprosy symptoms and visiting a medical doctor for these symptoms. This was labelled as patient delay. Participants were also asked to report the time that elapsed between their first visit to a medical doctor and them receiving a diagnosis of leprosy/Hansen’s disease. This was labelled as health system delay. Both time frames were recorded as ordinal variables to assist participant recall.

Other questions in the questionnaire consisted of variables potentially associated with patient delay and health-system delay. The questionnaire consisted of three sections:

1. **Demographics:** age, gender, employment, education, income and marital status.
2. **Variables specific to patient delay:** first symptom, age at symptom onset, who was informed about their symptoms and reason for waiting before consulting a doctor.
3. **Variables specific to health-system delay:** region, health centre funding, symptoms at presentation, number of symptoms, misdiagnosis and clinical examinations. The examinations considered in this study were those pertaining to the clinical diagnosis of leprosy. These included examination of the skin, sensory testing, nerve palpation and a skin smear/ biopsy sample.

Demographic variables and those specific to patient delay were analysed to determine associations of these factors with patient delay. Demographic variables and those specific to health system delay were analysed to determine associations with health-system delay.

All data were collected from the questionnaire. Where there was missing responses to the questionnaire, medical records were consulted to obtain this information if documented. All data obtained were anonymised.

### Statistical analysis

All data were treated and analysed using software *Statistical Package for Social Sciences* (SPSS) version 21.0.

Participants were grouped according to their responses to the questionnaire. Univariate analyses for patient delay and health system delay were conducted through Mann-Whitney U tests for binary predictor variables e.g. gender, Kruskal-Wallis tests for categorical predictor variables with more than two groups e.g. employment and spearman’s rank correlation for ordinal and continuous predictors e.g level of education and age at symptom onset, respectively. Multivariate analysis was conducted by ordinal regression and odds ratios calculated.

A p value of < 0.10 in the univariate analysis was the criterion for including variables in the multivariate analysis. After selection of variables for the regression models, those that lost significance were excluded. The statistical significance level considered for multivariate analyses was 5%, p <0.05.

Regarding the secondary aim of identifying factors that motivate patients to consult, patients were grouped according to their questionnaire responses to the question *‘What encouraged you to visit a medical doctor*?*’* Proportions of participants selecting each response were calculated.

### Sample description

After applying the exclusion criteria, 122 patients consented to participate in the study: 83 men and 39 women (Table 1). The age range for this sample was 20–86 years with a mean age of 48.9 years.

**Table 1:** Frequencies and percentages for gender, grade of disability and region of participants’ first consultation with a medical professional.

	Frequency (n)	Percentage (%)
**Gender**		
Male	83	68.0
Female	39	32.0
**Region of first consultation**		
Central-west	66	54.1
Southeast	43	27.3
Northeast	5	4.1
South	5	4.1
Missing	3	2.5

Income was classified according to the Brazilian institute of geography and statistics (IBGE), where three minimum salaries equals R$ 2,172, which is equivalent to £587 (Table 2) [33].

**Table 2:** Frequencies and percentages of participants’ personal and household incomes and highest level of education.

	Frequency (n)	Percentage (%)
**Personal income**		
No income	9	7.38
≤ 1 minimum salary	48	39.34
1–2 minimum salaries	38	31.15
2–3 minimum salaries	15	12.30
3–4 minimum salaries	4	3.28
4–5 minimum salaries	2	1.64
> 5 minimum salaries	3	2.46
Missing	3	2.46
**Household income**		
≤ 1 minimum salary	24	19.67
1–2 minimum salaries	37	30.33
2–3 minimum salaries	30	40.98
3–4 minimum salaries	14	11.48
4–5 minimum salaries	10	8.20
> 5 minimum salaries	6	4.92
Missing	1	0.82
**Highest level of Education**		
Never studied	6	4.92
Pre-school	9	7.38
Elementary school	58	47.54
Secondary school	16	13.11
High school	28	22.95
Higher education	5	4.10

## Results

Participants reported visiting health centres in both endemic and non-endemic regions of Brazil when they first consulted a doctor about their symptoms. Participants presented to doctors in four out of the five geographical regions of Brazil (Table 1). Sixty-six participants (45.3%) presented in the endemic central-west region of Brazil. Both patient and health-system delays were found to be independent of region in the univariate analyses using Kruskal-Wallis tests (X^2^ = 0.262, p = 0.967 and X^2^ = 4.982, p = 0.173 respectively).

### Patient delay

One hundred and twenty one participants, (99.2%), reported presenting to a medical doctor within 5 years of symptom onset and no patient delay exceeded 10 years (Table 3).

**Table 3:** Frequencies and cumulative percentages of presentation time and diagnosis time for participants in this sample.

Presentation time (symptom onset to doctor visit)	Frequency (n)	Cumulative percentage (%)	Diagnosis time (first consultation to diagnosis)	Frequency (n)	Cumulative percentage (%)
**0–2 weeks**	46	37.7	**0–2 weeks**	40	32.8
**15 days– 31 days**	16	50.8	**15 days– 31 days**	18	47.5
**1–3 months**	18	65.6	**1–3 months**	7	53.3
**3–6 months**	7	71.3	**3–6 months**	10	61.5
**6–12 months**	15	83.6	**6–12 months**	16	74.6
**1–2 years**	10	91.8	**1–2 years**	9	82.0
**3–5 years**	9	99.2	**3–5 years**	11	91.0
**6–10 years**	1	100	**6–10 years**	6	95.9
**>10 years**	0	100	**>10 years**	5*	100
**Total**	122	100	**Total**	122	100

* Diagnosis time ranged up to 41 years for one participant.

In the univariate analysis, patient delay was found to be independent of demographic variables (e.g. age, region and income) (p ≥ 0.10). Seven variables were found to be associated with patient delay, in univariate analysis (p < 0.1) and were included in the multivariate ordinal regression model (Table 4). Four factors remained significant at p < 0.05. Patient delay was found to have reduced over time, with the odds of participants presenting quicker, being 25.4% higher, for each decade that has passed since the 1930s (OR 0.746 95% CI 0.593–0.939, p = 0.013). For participants who thought their symptoms were due to leprosy but feared they would be isolated from their community, the odds of them waiting longer before consulting was ten times higher than those who did not fear isolation (OR 10.37, CI 2.18–49.45, p = 0.003). Fifty-five participants, (45.1%), said they waited before consulting because they did not believe their symptoms were serious. The odds of them waiting longer was three times higher compared to those who did consider their symptoms serious (OR 3.114, 95% CI 1.235–7.856, p = 0.016). Participants who visited a traditional healer before a qualified medical doctor had 10 times higher odds of waiting longer before consulting a trained medical doctor than those who did not (OR 10.386, CI 1.092–98.784, p = 0.042).

**Table 4:** Ordinal and logistic regression models for predictors of longer presentation time and delayed presentation respectively.

Predictors of longer patient delay, Pseudo R-square = 0.368							
Variable	Coefficient	Standard error (S.E).	z	P value	Odds ratio (OR) Exp(B)	95% Conf. Interval for Exp(B)	
						Lower	Upper
*Feared isolation	2.339	0.7969	8.614	.003*	10.371	2.175	49.448
*Decade of first Symptom (earliest decade (1930s) as reference)	-.293	0.1174	6.225	.013*	0.746	.593	.939
*Thought symptoms were not serious	1.136	0.4721	5.790	.016*	3.114	1.235	7.856
*Visited a natural healer	2.340	1.1493	4.147	.042*	10.386	1.092	98.784
**Expected symptoms would disappear**	.871	1.831	3.159	.076	2.389	.914	6.241
**Told no-one about symptoms**	.588	0.4680	1.578	.209	1.800	.719	4.505
**Had no pain**	.443	0.4585	.933	.334	1.557	.634	3.825

All variables included in these regression models were those, which were found to be significantly associated with presentation time and delayed presentation at p < 0.1 in univariate analysis.

* Variables achieving significance at p = 0.05 in regression

### Health system delay

Five participants reported receiving a diagnosis of leprosy more than 10 years after consulting a medical doctor about their symptoms. Health-system delay ranged up to 41 years for one participant (Table 3).

No statistically significant correlation between the number of presenting symptoms and health-system delay (r_s_ 0.036, p = 0.698) was found in univariate analysis with Spearman’s rank correlation. In the multivariate analyses, health-system delay was found to be independent of demographic variables (e.g. age and region of consultation) (p ≥ 0.05).

Reported misdiagnosis remained statistically significantly associated with a longer health-system delay in multivariate analysis (Table 5). The likelihood of misdiagnosed patients receiving a later diagnosis was nearly three times higher than those who were not misdiagnosed (those who were either correctly diagnosed or not given any diagnosis on their first visit) (OR 2.867, 95% CI 1.288–6.384, p = 0.01). The number of examinations performed by doctors on the participants’ first consultation was associated with health system delay. For every additional examination performed, the odds of a patient receiving a quicker diagnosis of leprosy was 46% (OR 0.539, CI 0.393–0.739). The only presenting symptom associated with health system delay was hypo-pigmented, insensitive skin lesions. Patients presenting with insensitive skin lesions had a 55% higher chance of being diagnosed quicker than those who did not present with this symptom (OR 0.463, CI 0.222–0.964, p = 0.039).

**Table 5:** Ordinal and logistic regression models for predictors of longer diagnosis time and delayed diagnosis respectively.

Predictors of longer health-system delay, Pseudo R-square = 0.445							
Variable	Coefficient	Standard error S.E.	z	P value	Odds ratio OR Exp(B)	95% Conf. Interval for Exp(B)	
						Lower	Upper
*Number Of Exams (0 as reference)	-0.618	0.1609	14.758	<0.001	0.539	0.393	0.739
*Misdiagnosed	-1.053	0.4083	6.656	0.010	2.867	1.288	6.384
*Presented with insensitive skin lesion	0.770	0.3740	4.240	0.039	0.463	0.222	0.964
**Gender** (female as reference)	-0.695	0.3752	3.434	0.064	0.499	0.239	1.041
**Decade of presentation** (earliest decade 1940 as reference)	-0.175	0.1068	2.675	0.102	0.840	0.681	1.035
**Referred to another doctor**	-0.511	0.3777	1.828	0.176	1.667	0.795	3.494
**Examined**	0.256	0.5231	0.240	0.624	0.774	0.278	2.158

All variables included in these regression models were those, which were found to be significantly associated with diagnosis time and delayed diagnosis at p < 0.1 in univariate analysis.

*Variables achieving significance at p = 0.05 in the regression mode

Fifty-two participants (42.6%) reported being misdiagnosed. Of these, 17.3% reported being misdiagnosed with skin allergy and 13.5% with other skin conditions such as ringworm and furunculosis. Another 13.5% reported a diagnosis of rheumatism and 9.6% of vascular conditions including infarct (occlusion of blood supply).

### Motivators to consulting

The majority of participants answered that they first visited a doctor, due to their symptoms worsening, (48.4%), or persisting (20.5%).

## Discussion

The aims of this study were achieved as we identified factors associated with patient and health system delays that could be contributing to the overall delayed diagnosis of leprosy in Brazil. The principal factors found to be associated with patient delay were participants who feared community isolation and those who visited a traditional healer. The odds of each waiting longer before consulting a medical doctor was ten times higher compared to those who did not. However, these only constituted a small proportion of participants. Many more participants delayed because they did not think their symptoms were serious. The odds of these waiting longer before consulting was three times greater compared to those who did believe their symptoms were serious. These three factors explained 37.0% of the variance in patient delay and therefore, could help reduce patient delay, if targeted.

These findings indicate that stigma towards leprosy sufferers remains despite the availability of a cure, even in endemic regions [12,13]. Fear of isolation was a strong predictor of longer patient delay, implying that community isolation still exists in Brazil, despite compulsory confinement ending in 1962. Some patients seem to conceal their symptoms and delay in seeking treatment due to fear of social exclusion [32]. Yet only 8.2% of participants delayed due to the fear of isolation. This is likely due to governmental efforts to abolish this stigma; by integrating leprosy care into primary care and changing its name from leprosy to Hansen’s disease. Nonetheless, political commitment to diminish the stigma surrounding the disease must persist [13,34]. Through national health programmes, the public should be informed that leprosy is curable and that the disability, which has instigated much fear is preventable [14,31,35].

Many patients appear to still be ignorant regarding leprosy symptoms. Nearly half of the participants in this study (45.1%) waited before consulting a doctor because they did not believe their symptoms were serious. This was supported by the observation that 68.9% of participants consulted a doctor because their symptoms either persisted or worsened. These findings support research in India and suggest an important need to educate patients regarding the early symptoms of leprosy, in order to encourage them to present early [1,5,13]. As leprosy is a disease of the poor and literacy rates are low among this population in Brazil, it could be useful to incorporate graphics or animations into patient education tools from primary care clinics or even in public areas such as bus stops, where educational posters could be displayed [3,36].

Visiting a traditional healer was not found to be as clinically relevant as found in Nepal, Nigeria and Ethiopia, in contributing to patient delay [14,37,38]. Although statistically significant, only a small proportion (2.5%) of participants in this study reported visiting a traditional healer. This percentage may be higher in poorer, more rural areas where access to healthcare is limited; future research should be done in such areas to explore this further [28].

The principal association with longer health system delays was found to be patient reported misdiagnosis. Higher numbers of clinical examinations (pertaining to leprosy diagnosis) performed by doctors were associated with a quicker diagnosis, whilst patients presenting to a doctor with an insensitive skin lesion were diagnosed quicker than those without this symptom. These factors together explain 44.5% of the variance in health-system delay. Acting on these factors could help to reduce the health-system delay in leprosy diagnosis.

High rates of misdiagnosis of leprosy patients have previously been reported in Brazil where 32.5% of participants were misdiagnosed [10]. Similarly, in this study, 42.6% reported being misdiagnosed. Participants commonly reported being misdiagnosed with conditions such as rheumatism and skin allergy. This possible lack in clinician suspicion could be explained by the decentralisation of leprosy care in Brazil. However, despite the gradual decline in the incidence of leprosy cases in Brazil, health professionals should expect to encounter leprosy patients during their practice, especially in endemic regions. Medical students must also continue to be educated regarding leprosy symptoms at university [28]. The only symptom associated with a quicker diagnosis was the cardinal symptom of an insensitive skin lesion. Health system delay was found to be independent to the number of symptoms patients presented to doctors with. Clinicians may therefore benefit from further education regarding leprosy symptoms other than insensitive skin lesions. Primary health clinics could trial various educational tools to assist doctors in differentiating between leprosy symptoms and those of other diseases e.g. rheumatism and skin allergy. This could be done through Internet learning where possible or education leaflets in resource poor settings. Research could be done to determine their effectiveness at increasing clinicians’ suspicions of leprosy and reducing health system delays in diagnosis. In light of these findings, future qualitative and quantitative research could also explore primary care clinicians' knowledge of leprosy and its symptoms.

Limitations of this study include accuracy of recall and social acceptability bias. Although measures were taken within the questionnaire to assist participant recall, data collection largely depended on participant reported information. Furthermore, a sizeable proportion of participants were functionally illiterate and required verbal questionnaire delivery. These responses may have been influenced by social acceptability bias with participants reporting shorter patient delays. Nevertheless, as high illiteracy rates occur among leprosy patients, it was necessary to include illiterate participants in this study in order to provide a more representative sample of leprosy patients [3,39].

This study was limited to three referral centres. This sample is likely to differ from simple leprosy cases, which are dealt with in the community and results from this study may not be generalizable throughout the country. Although delays were found to be independent of region in this study, previous research has indicated that reason for delay can vary between regions of the same country, with some areas seeing long health system delays whilst others seeing longer patient delays [30]. Few participants in this study had presented to health centres in the rural, endemic north and northeast regions of Brazil which have reported some of the highest rates of leprosy but low availability of human recourses and limited access to health care [28, 40]. Therefore similar research exploring the delay in diagnosis of leprosy in these regions would be of great value.

In conclusion, this study highlights the potential need for further patient education regarding disease symptoms and the reduction of stigma to encourage patients to seek earlier medical care. Furthermore, this study supports the high rates of misdiagnosis amongst leprosy patients reported elsewhere in Brazil and suggests the need for greater education of primary care clinicians with regards to leprosy symptoms. Exploring primary care clinicians’ knowledge of disease symptoms and trialling educational tools could assist in reducing the delay in diagnosis and continued transmission of leprosy in Brazil. Finally, similar research to this study should explore the delay in diagnosis of leprosy in northern regions of Brazil.

## Supporting Information

S1 Appendix Quantitative questionnaire: Data collection tool. The questionnaire consists of three sections: 1. Demographics: age, gender, employment, education, income and marital status. 2. Variables specific to patient delay: first symptom, age at symptom onset, who was informed about their symptoms and reason for waiting before consulting a doctor. 3. Variables specific to health-system delay: region, health centre funding, symptoms at presentation, number of symptoms, misdiagnosis and clinical examinations. The examinations considered were those pertaining to the clinical diagnosis of leprosy and included examination of the skin, sensory testing, nerve palpation and a skin smear/ biopsy sample.
(PDF)

## References

[1] Lockwood DN, Suneetha S. Leprosy: Too complex a disease for a simple elimination paradigm. Bull World Health Organ. 2005; 83: 230–235. 15798849 PMC2624210

[2] Hotez PJ, Bottazzi ME, Franco-Paredes C, Ault SK, Periago MR. The neglected tropical diseases of Latin America and the Caribbean: A review of disease burden and distribution and a roadmap for control and elimination. PLoS Neglected Tropical Diseases. 2008; 2: 300. 10.1371/journal.pntd.0000300 PMC2553488 18820747

[3] Kerr-Pontes LR, Barreto ML, Evangelista CM, Rodrigues LC, Heukelbach J, Feldmeier H. Socioeconomic, environmental, and behavioural risk factors for leprosy in north-east Brazil: Results of a case-control study. Int J Epidemiol. 2006; 35: 994–1000. 16645029 10.1093/ije/dyl072

[4] van Brakel WH, Sihombing B, Djarir H, Beise K, Kusumawardhani L, Yulihane R, et al Disability in people affected by leprosy: The role of impairment, activity, social participation, stigma and discrimination. Global Health Action. 2012; 5 Available: http://www.ncbi.nlm.nih.gov/pmc/articles/PMC3402069/10.3402/gha.v5i0.18394 PMC3402069 22826694

[5] Cross H. The prevention of disability for people affected by leprosy: Whose attitude needs to change? Lepr Rev. 2007; 78: 321–329. 18309705

[6] World Health Organization (WHO). Weekly Epidemiological Record, Geneva, Switzerland. Global leprosy situation, 2012. WHO. Weekly Epidemiological Record 87: 317–328.22919737

[7] Pannikar V. Enhanced global strategy for further reducing the disease burden due to leprosy: 2011–2015. Lepr Rev. 2009; 80: 353–354. 20306634

[8] Lana FF, Fabri AC, Lopes FN, Carvalho AM, Lanza FM. Deformities due to leprosy in children under fifteen years old as an indicator of quality of the leprosy control programme in Brazilian municipalities. Journal of Tropical Medicine 2013 Available: http://dx.doi.org/10.1155/2013/812793 10.1155/2013/812793 PMC3614053 23577038

[9] Alencar CH, Ramos AN Jr, Barbosa JC, Kerr LR, De Oliveira M, Heukelbach J. Persisting leprosy transmission despite increased control measures in an endemic cluster in Brazil: The unfinished agenda. Lepr Rev. 2012; 83: 344–353. 23614252

[10] Souza CS, Bacha JT. Delayed diagnosis of leprosy and the potential role of educational activities in Brazil. Lepr Rev. 2003; 74: 249–258. 14577470

[11] Deps PD, Guedes BV, Filho J, Andreatta MK, Marcari RS, Rodrigues LC. Delay in the diagnosis of leprosy in the metropolitan region of Vitória, Brazil. Lepr Rev. 2006; 77: 41–47. 16715689

[12] Zhang F, Chen S, CHU T. Healthcare seeking behaviour and delay in diagnosis of leprosy in a low endemic area of China. Lepr Rev. 2009; 80: 416–423. 20306640

[13] Samraj A, Kaki S, Rao P. Help-seeking habits of untreated leprosy patients reporting to a referral hospital in Uttar Pradesh, India. Indian J Lepr. 2012; 84: 123 23236699

[14] Choulagai B, Onta S, Bhattarai P. Patient delay in leprosy treatment in Jhapa district Nepal. Journal of Nepal Health Research Council. 2005; 3: 29–34.

[15] Nicholls P, Wiens C, Smith W. Delay in presentation in the context of local knowledge and attitude towards leprosy-the results of qualitative fieldwork in Paraguay. International Journal of Leprosy and Other Mycobacterial Diseases. 2003; 71: 198–209. 14608815 10.1489/1544-581X(2003)71<198:DIPITC>2.0.CO;2

[16] Bainson K, Van den Borne B. Dimensions and process of stigmatization in leprosy. Lepr Rev. 1998; 69: 341–350. 9927806 10.5935/0305-7518.19980034

[17] Deepak S. Answering the rehabilitation needs of leprosy-affected persons in integrated setting through primary health care services and community-based rehabilitation. Indian J Lepr. 2003; 75: 127–146. 15255400

[18] Tekle-Haimanot R, Forsgren L, Gebre-Mariam A, Abebe M, Holmgren G, Heijbel J, et al Attitudes of rural people in central Ethiopia towards leprosy and a brief comparison with observations on epilepsy. Lepr Rev. 1992; 63: 157–168. 1640784 10.5935/0305-7518.19920021

[19] Van Brakel WH. Measuring leprosy stigma-a preliminary review of the leprosy literature. International Journal of Leprosy and Other Mycobacterial Diseases. 2003; 71: 190–197. 14608814 10.1489/1544-581X(2003)71<190:MLSPRO>2.0.CO;2

[20] Weiss MG, Ramakrishna J. Stigma interventions and research for international health. The Lancet. 2006; 367: 536–538. 10.1016/S0140-6736(06)68189-0 16473134

[21] Rinaldi A. The global campaign to eliminate leprosy. PLoS medicine. 2005; 2: 341. 10.1371/journal.pmed.0020341 PMC1322279 16363908

[22] John AS, Raju M, Rao P. A case of stigma in an urban metropolis in India. What new tools should be used? Lepr Rev. 2013; 84: 92–94. 23741886

[23] Nations MK, Lira GV, Catrib AMF. Stigma, deforming metaphors and patients' moral experience of multibacillary leprosy in Sobral, Ceará state, Brazil. Cadernos de Saúde Pública 2009; 25: 1215–1224. 19503952 10.1590/s0102-311x2009000600004

[24] Fuzikawa PL, Acurcio FDA, Velema JP, Cherchiglia ML. Factors which influenced the decentralisation of leprosy control activities in the municipality of Betim, Minas Gerais state, Brazil. Lepr Rev. 2010; 81: 196 21067060

[25] Maciel E, Golub J, Peres R, Hadad D, Fávero J, Molino L, et al Delay in diagnosis of pulmonary tuberculosis at a primary health clinic in Vitória, Brazil. The International Journal of Tuberculosis and Lung Disease. 2010; 14: 1403–1410. 20937179 PMC3697918

[26] Sichieri R, Coitinho DC, Leao MM, Recine E, Everhart JE. High temporal, geographic, and income variation in body mass index among adults in Brazil. Am J Public Health. 1994; 84: 793–798. 8179051 10.2105/ajph.84.5.793 PMC1615023

[27] de Queiroz, ML. A Hanseníase no estado de Mato Grosso. Dissertation, universidada federal de Mato Grosso, 2009. Available: http://www.saude.mt.gov.br/upload/documento/97/dissertacao-de-maria-de-lourdes-de-queiroz-a-hanseniase-no-estado-de-mato-grosso-[97-021209-SES-MT].pdf.

[28] Penna MLF, De Oliveira M, Penna G. The epidemiological behaviour of leprosy in Brazil. Lepr Rev. 2009; 80: 332 19961107

[29] Imbiriba EB, Hurtado-Guerrero JC, Garnelo L, Levino A, Cunha MdG, Pedrosa V, et al Epidemiological profile of leprosy in children under 15 in Manaus (northern Brazil), 1998–2005. Rev Saude Publica. 2008; 42: 1021–1026. 19031534 10.1590/s0034-89102008005000056

[30] De Rojas V, Hernández O, Gil R. Some factors influencing delay in leprosy diagnosis. Bulletin-pan American Health Organization. 1994; 28: 156–156. 8069335

[31] Tsutsumi A, Izutsu T, Islam A, Maksuda A, Kato H, Wakai S, et al The quality of life, mental health, and perceived stigma of leprosy patients in Bangladesh. Soc Sci Med. 2007; 64: 2443–2453. 17382441 10.1016/j.socscimed.2007.02.014

[32] Nunes JM, Oliveira EN, Vieira NF. Ter hanseníase: Percepções de pessoas em tratamento. Revista da Rede de Enfermagem do Nordeste-Rev Rene. 2012; 9: 99–104.

[33] Instituto Brasileiro de Geografia. Síntese de indicadores sociais Uma análise das condições de vida da população Brasileira, IBGE: 2013.

[34] Lesshafft H, Heukelbach J, Barbosa JC, Rieckmann N, Liesenfeld O, Felmeier H, et al Perceived social restriction in leprosy-affected inhabitants of a former leprosy colony in northeast Brazil. Lepr Rev. 2010; 81: 69 20496571

[35] Monteiro YN. Prophylaxis and exclusion: Compulsory isolation of Hansen's disease patients in São Paulo. História, Ciências, Saúde-Manguinhos. 2003;10: 95–121. 14650408 10.1590/s0104-59702003000400005

[36] Mayeaux EJ, Murphy PW, Arnold C, Davis TC, Jackson RH, Sentell T. Improving patient education for patients with low literacy skills. Am Fam Physician. 1996; 53: 205–211. 8546047

[37] Amenu A, Nash J, Tamiru T, Byass P. Patterns of health-seeking behaviour amongst leprosy patients in former Shoa province, Ethiopia. Ethiopian Journal of Health Development. 2000; 14: 43–47.

[38] van de Weg N, Post EB, Lucassen R, de Jong JT, Van den Broek J. Explanatory models and help-seeking behaviour of leprosy patients in Adamawa state, Nigeria. Lepr Rev. 1998; 69: 382–389. 9927811 10.5935/0305-7518.19980039

[39] Monteiro CA, Benicio MH, Conde WL, Konno S, Lovadino AL, Barros A, et al Narrowing socioeconomic inequality in child stunting: The Brazilian experience, 1974–2007. Bull World Health Organ. 2010; 88: 305–311. doi: 10.2471/BLT.09.069195 20431795 10.2471/BLT.09.069195 PMC2855601

[40] Hungria EM, Oliveira RM, Souza AL, Costa MB, Souza VN, Silva EA, et al Seroreactivity to new Mycobacterium leprae protein antigens in different leprosy-endemic regions in Brazil. Memórias do Instituto Oswaldo Cruz. 2012 12; 107:104–11. 23283461 10.1590/s0074-02762012000900017

