# Supplementary material for: Factors Contributing to the Delay in Diagnosis and Continued Transmission of Leprosy in Brazil – An Explorative, Quantitative, Questionnaire Based Study
Source: PLoS Negl Trop Dis. 2016 Mar 15;10(3):e0004542. doi: 10.1371/journal.pntd.0004542 (PMC4792453; doi:10.1371/journal.pntd.0004542)
Supplement: S1 Appendix — The questionnaire consists of three sections: 1. Demographics: age, gender, employment, education, income and marital status. 2. Variables specific to patient delay: first symptom, age at symptom onset, who was informed about their symptoms and reason for waiting before consulting a doctor. 3. Variables specific to health-system delay: region, health centre funding, symptoms at presentation, number of symptoms, misdiagnosis and clinical examinations. The examinations considered were those pertaining to the clinical diagnosis of leprosy and included examination of the skin, sensory testing, nerve palpation and a skin smear/ biopsy sample. (PDF) [file pntd.0004542.s001.pdf]

Appendix

Thank you very much for agreeing to complete this questionnaire.
Your involvement in this study is greatly appreciated and will help us to understand more about leprosy and the effects of this disease.

Please try and answer all questions as truthfully as possible - all answers will remain completely anonymous.

Please complete the following questions:
Fill in the blank spaces or where there is more than one option; please tick the correct boxes (as seen in the example below):

Example: ☑ Yes ☐ No

1) Age: ______________________________

2) Gender: ☐ Male ☐ Female

3) In which city and in which state do you live?

   ____

4) Current Employment Status:

   ☐ Unemployed
   ☐ Part-time work
   ☐ Full-time work
   ☐ Self-employed
   ☐ Retired

5) Occupation: ____

6) Highest Level of Education received:

   ☐ Never studied
   ☐ Pre-school Education
   ☐ Primary School I (years 1-5)
   ☐ Primary School II (years 6-9)
   ☐ Secondary School
   ☐ Higher education

7) Marital Status:

- ☐ Single
- ☐ Married
- ☐ In a Relationship
- ☐ Living with Partner
- ☐ Separated
- ☐ Divorced
- ☐ Widowed

8) Number of children:

☐ 0	☐ 4
☐ 1	☐ 5
☐ 2	☐ 6
☐ 3	☐ 7+

9) Who do you currently live with?

- ☐ Alone
- ☐ With a partner/spouse
- ☐ With children
- ☐ With partner/spouse and children
- ☐ With other family
- ☐ Other *Please specifiy:*

____

____

10) Personal Income:

- ☐ Less than 1 minimum salary
- ☐ 1 minimum salary
- ☐ 1-2 minimum salaries
- ☐ 2-3 minimum salaries
- ☐ 3-4 minimum salaries
- ☐ 4-5 minimum salaries
- ☐ Other ________________

11) Household Income:

- ☐ Less than 1 minimum salary
- ☐ 1 minimum salary
- ☐ 1-2 minimum salaries
- ☐ 2-3 minimum salaries
- ☐ 3-4 minimum salaries
- ☐ 4-5 minimum salaries
- ☐ Other ________________

12) How old were you when you received a diagnosis of leprosy? In what year was this?

____

13) Does anybody else in your family have leprosy? ☐ Yes ☐ No

14) Apart from leprosy, do you have any other illnesses? ☐ Yes ☐ No

If yes, what?

____

____

Section 2

Before starting this section, please take time to try and remember back to when you first noticed symptoms for leprosy. Think about the time of year it was and how old you were to help you remember. I'd like you to remember what you thought at the time.

1. In what month and year did you first experience symptoms to do with Hansen's disease?

   ____

2. How old were you when you first experienced this symptom?

   ____

3. What were the first symptoms you noticed? (Choose only 1 option)

   ☐ Pale patches on your skin

   ☐ Pale patches on your skin with no sensation

   ☐ Lumpy or thickened skin

   ☐ Runny nose or nose bleed

   ☐ Difficulty seeing

   ☐ Pain or tingling in your arms, legs, hands, feet or around eyes

   ☐ Loss of feeling on patches of skin or hands or feet

   ☐ Muscle weakness in your, hands feet, arms or legs (difficulty moving them)

   ☐ Muscle weakness around your eyes (difficult to close eyes tightly)

   ☐ Cuts, wounds or ulcers

   ☐ Other (*please specify*) ________________________________

4. a) Had you heard about Hansen's disease at that time?

   ☐ Yes ☐ No

b) If you hadn't heard about Hansen's disease, what about leprosy? Had you heard about leprosy?

☐ Yes ☐ No

5. Did you think that your symptoms could be due to Hansen's disease or leprosy?

☐ Yes ☐ No

6. Some people like to tell others soon after they experience symptoms (within 2 weeks). Did you do this? (You can select more than one option)

☐ I did not tell anyone

☐ Family member or friend

☐ Priest

☐ Any other religious leader

☐ Local healer

☐ I went straight to a medical practitioner

☐ Other *(please specify)* ____

7. At the time you noticed your first symptoms, roughly how far away was the nearest health centre from your home?

☐ 0 – 1 km ☐ 5 – 10 km
☐ 1 – 3 km ☐ 10 – 20 km
☐ 3 – 5 km ☐ > 20 km

8. Many people wait a while before visiting a medical practitioner about their symptoms. How long did you wait before you tried seeing a medical doctor about your symptoms?

☐ 0 – 2 weeks ☐ 3 - 6 months (including 6 months)
☐ 15 days – 4 weeks (1 month) ☐ 6 months – 1 year (including 1 year)
☐ 1 – 3 months (including 3 months) ☐ More than 1 year. How many?_____

9. Why was it that you waited before seeing a medical practitioner? (you can select more than one option)

- ☐ I did not wait, I went straight away
- ☐ I lived too far away from a health centre
- ☐ I couldn’t get an appointment
- ☐ I couldn’t afford to pay for an appointment
- ☐ I could not afford to take time off work to visit a health centre
- ☐ I did not think my symptoms were serious
- ☐ I thought my symptoms would go away on their own
- ☐ I was not in pain
- ☐ Family member/friend told me not to tell anyone
- ☐ I was afraid it was something serious
- ☐ I was afraid it might be Hansen’s disease but didn’t want anyone to know
- ☐ I thought it was Hansen’s disease but didn’t want to be isolated from my community
- ☐ I knew it was Hansen’s disease but did not think anything could be done to help
- ☐ Other (*please specify*) ____

10. Was there anyone else in your household with known Hansen’s disease at the time?

☐ Yes ☐ No

The next questions are about when you made contact with the health service and about your first consultation:

11. a) What year was it when you visited a doctor (medical practitioner) for the first time for your symptoms?

    ____

    b) How old were you when you visited a doctor (medical practitioner) for the first time for your symptoms?

    ____

12. Where? Please state the city/ state that you were in and the health centre.

    ____

13. What encouraged you to go to a health centre and see a medical doctor? (You can select more than one option)

    - ☐ My symptoms didn’t go away
    - ☐ My symptoms got worse
    - ☐ My symptoms got so bad I couldn’t hide the condition anymore
    - ☐ A friend or family member encouraged me to go
    - ☐ My local healer told me to visit a medical doctor
    - ☐ The treatment I was using was not working
    - ☐ I was visiting the doctor for another reason and I mentioned it whilst I was there.
    - ☐ I was visiting the doctor for another reason and they noticed my symptoms.
    - ☐ Other *(please specify)*:

      ____

      ____

14. a) What symptoms were you experiencing when you first saw a medical doctor? (More than one can be selected)

- ☐ Pale patches on your skin
- ☐ Pale patches on your skin with no sensation
- ☐ Lumpy or thickened skin
- ☐ Runny nose or nose bleed
- ☐ Difficulty seeing
- ☐ Pain or tingling in your arms, legs, hands, feet or around eyes
- ☐ Loss of feeling on patches of skin or hands or feet
- ☐ Muscle weakness in your, hands feet, arms or legs (difficulty moving them)
- ☐ Muscle weakness around your eyes (difficult to close eyes tightly)
- ☐ Cuts, wounds or ulcers
- ☐ Other (*please specify*) ________________________________

b) Which symptom were you most concerned about out of all your symptoms? (Select only the main symptom)

- ☐ Pale patches on your skin
- ☐ Pale patches on your skin with no sensation
- ☐ Lumpy or thickened skin
- ☐ Runny nose or nose bleed
- ☐ Difficulty seeing
- ☐ Pain or tingling in your arms, legs, hands, feet or around eyes
- ☐ Loss of feeling on patches of skin or hands or feet
- ☐ Muscle weakness in your, hands feet, arms or legs (difficulty moving them)
- ☐ Muscle weakness around your eyes (difficult to close eyes tightly)
- ☐ Cuts, wounds or ulcers
- ☐ Other (*please specify*) ________________________________

15. Did the doctor suspect that you might have Hansen's disease?

☐ Yes ☐ No

16. Did the doctor examine you?

☐ Yes ☐ No (if you selected 'No', please skip to question 18)

17. Did the doctor perform any of these examinations on you? (On your first visit)

☐ They looked at my skin

☐ They tested the feeling in my skin (often done over a pale skin patch)

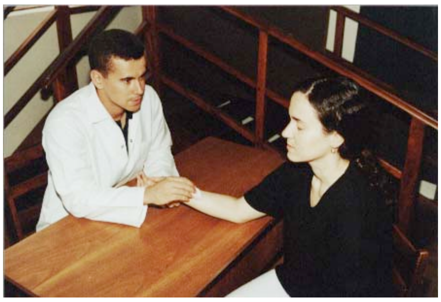

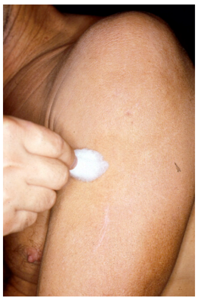

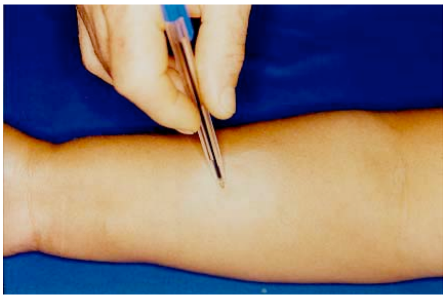

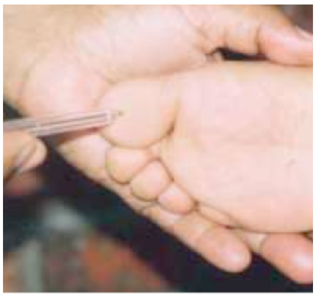

☐ They felt my nerves (e.g. at the elbow or in my legs)

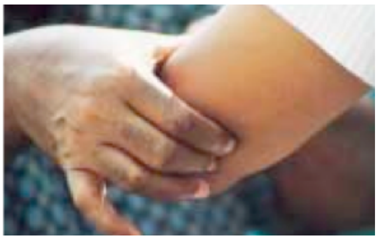

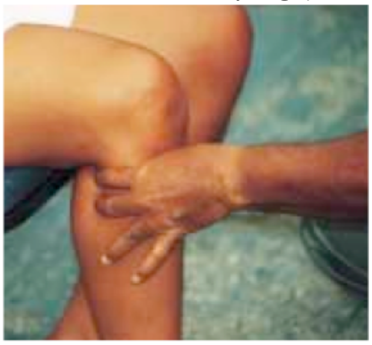

☐ They took a sample of skin (cut made in skin usually at the earlobe or arm)

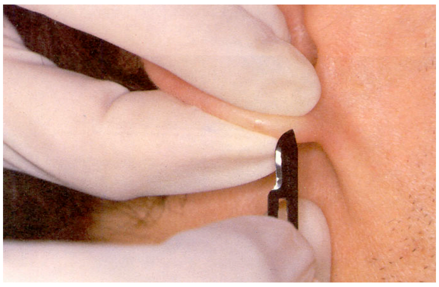

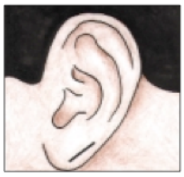

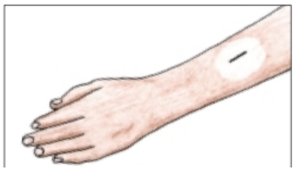

18. Did the doctor diagnose you with leprosy on this first visit?

- ☐ Yes (If you marked this option, you do not need to continue answering the questionnaire)
- ☐ No

19. It is easy for Hansen's disease to be mistaken for a different condition. Did your doctor diagnose you with another medical condition instead? (Please only select one option).

- ☐ No
- ☐ Bone condition
- ☐ Nerve condition
- ☐ Blood vessel condition
- ☐ Other (*please specify*) ____

20. Did your doctor refer you to another doctor?

☐ Yes ☐ No

23. How many different doctors did you see before being diagnosed with leprosy? (Including the doctor that diagnosed you)

- ☐ 1
- ☐ 2-3
- ☐ 4-5
- ☐ More than 5

21. When were you diagnosed with Hansen's disease (leprosy)? (Please give month and year if possible)

____

22. How long after your first visit to a health centre were you diagnosed with leprosy?

☐ 0 – 2 weeks	☐ 3 - 6 months (including 6 months)
☐ 15 days – 4 weeks (1 month)	☐ 6 months – 1 year (including 1 year)
☐ 1 – 3 months (including 3	☐ months) More than 1 year. How many?_____
